# Three novel oligosaccharides synthesized using *Thermoanaerobacter brockii *kojibiose phosphorylase

**DOI:** 10.1186/1752-153X-1-18

**Published:** 2007-06-28

**Authors:** Natsuko Takahashi, Eri Fukushi, Shuichi Onodera, Noureddine Benkeblia, Tomoyuki Nishimoto, Jun Kawabata, Norio Shiomi

**Affiliations:** 1 Department of Food and Nutrition Sciences, Graduate School of Dairy Science Research, Rakuno Gakuen University, Ebetsu 069-8501, Japan; 2 Graduate School of Agriculture, Hokkaido University, Sapporo 060-8589, Japan; 3 Hayashibara Biochemical Laboratories, Inc., Okayama 702-8006, Japan

## Abstract

**Background:**

Recently synthesized novel oligosaccharides have been produced primarily by hydrolases and glycosyltransferases, while phosphorylases have also been subject of few studies. Indeed, phosphorylases are expected to give good results via their reversible reaction. The purpose of this study was to synthesis other novel oligosaccharides using kojibiose phosphorylase.

**Results:**

Three novel oligosaccharides were synthesized by glucosyltransfer from β-D-glucose 1-phosphate (β-D-G1P) to xylosylfructoside [*O*-α-D-xylopyranosyl-(1→2)-β-D-fructofuranoside] using *Thermoanaerobacter brockii *kojibiose phosphorylase. These oligosaccharides were isolated using carbon-Celite column chromatography and preparative high performance liquid chromatography. Gas liquid chromatography analysis of methyl derivatives, MALDI-TOF MS and NMR measurements were used for structural characterisation. The ^1^H and ^13^C NMR signals of each saccharide were assigned using 2D-NMR including COSY (correlated spectroscopy), HSQC (herteronuclear single quantum coherence), CH_2_-selected E-HSQC (CH_2_-selected Editing-HSQC), HSQC-TOCSY (HSQC-total correlation spectroscopy) and HMBC (heteronuclear multiple bond correlation).

**Conclusion:**

The structure of three synthesized saccharides were determined, and these oligosaccharides have been identified as *O*-α-D-glucopyranosyl-(1→2)-*O*-α-D-xylopyranosyl-(1→2)-β-D-fructofuranoside (saccharide **1**), *O*-α-D-glucopyranosyl-(1→2)-*O*-α-D-glucopyranosyl-(1→2)-*O*-α-D-xylopyranosyl-(1→2)-β-D-fructofuranoside (saccharide **2**) and *O*-α-D-glucopyranosyl-(1→[2-*O*-α-D-glucopyranosyl-1]_2_→2)-*O*-α-D-xylopyranosyl-(1→2)-β-D-fructofuranoside (saccharide **3**).

## Background

The synthesis of oligosaccharides with various functions has been actively performed for some time. Such oligosaccharides are primarily synthesized by hydrolases and glycosyltransferases. Although phosphorylases have been the subject of few studies, they are expected to give good results via their reversible reaction.

*Thermoanaerobacter brockii *kojibiose phosphorylase is known to catalyze the reversible phosphorolysis of kojibiose into β-D-glucose 1-phosphate (β-D-G1P) and glucose [1]. In this reversible reaction, the enzyme uses various oligosaccharides as accepters. Furthermore, this enzyme is understood to catalyze glucosyltransfer from β-D-G1P to position 2 of the glucose residue in oligosaccharides such as raffinose, stachyose [2], 1-kestose, nystose [3] and sucrose.

Xylosylfructoside [*O*-α-D-xylopyranosyl-(1→2)-β-D-fructofuranoside] is an oligosaccharide that exhibits non-cariogenicity and selective growth stimulation in bifidobacteria. This oligosaccharide does not exist naturally, but can be synthesized by levansucrases from *Bacillus subtilis *[4] and *Aerobacter levanicus *[5] or β-fructofuranosidases from *Penicillum sp*. *K-25 *[6], *Penicillum frequertans T-1*[7] and *Scopulariopsis brevicaulis *[8].

In this paper we report when xylosylfructoside is used as a substrate, *Thermoanaerobacter brockii *kojibiose phosphorylase catalyzes glucosyltransfer from β-D-G1P to position 2 of the xylose residue. However transfer to other saccharides lacking glucose residues does not occur, with the exception of sorbose.

Here, we succeed in the synthesis of three new oligosaccharides, saccharides **1**, **2 **and **3**, by glucosyltansfer from β-D-G1P to xylosylfructoside using *Thermoanaerobacter brockii *kojibiose phosphorylase.

We also carried out structural analysis of the synthesized oligosaccharides using NMR spectroscopy. Structural analysis using NMR of the saccharides with a high degree of polymerization by NMR is now becoming a standard technique. However, it is difficult to assign the proton (^1^H) and carbon (^13^C) signals in oligosaccharides whose residues are similar, particularly in oligosaccharides with numerous methylene (CH_2_) groups, such as fructooligosaccharides and kojioligosaccharides.

The purpose of this study is to synthesize three novel oligosaccharides by kojibiose phosphorylase and carry out the full assignment of the ^1^H and ^13^C signals using 2D-NMR techniques such as COSY, HSQC, CH_2 _E-HSQC, HSQC-TOCSY and HMBC.

## Results and discussion

### Oligosaccharide synthesis and identification

**Figure 1:**
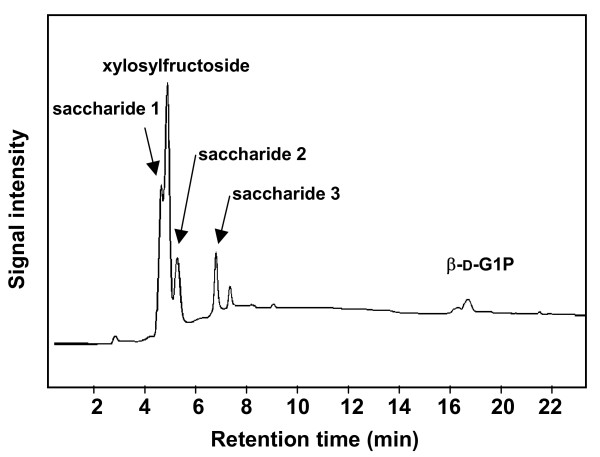
HPAEC of saccharides produced from xylosylfructoside and β-D-G1P using kojibiose phosphorylase. The enzyme reaction was carried out with 0.12 M xylosylfructoside and 0.10 M β-D-G1P at 50°C for 54 h.

Saccharides **1**, **2 **and **3 **were synthesized from xylosylfructoside [*O*-α-D-xylopyranosyl-(1→2)-β-D-fructofuranoside] and β-D-G1P using kojibiose phosphorylase. The HPAEC chart of saccharides **1**, **2 **and **3 **synthesized after 54 h reaction is shown in Figure 1. From the reaction mixture, saccharides **1**, **2 **and **3 **were isolated by successive chromatographic procedures using carbon-Celite and ODS columns, and finally obtained as a white powder.

Saccharides **1**, **2 **and **3 **were shown to be homogenous using HPAEC [*t*_R_, retention time of sucrose = 1.00; 1.51, 1.80 and 2.35]. The physical value [α]20D
 MathType@MTEF@5@5@+=feaafiart1ev1aaatCvAUfKttLearuWrP9MDH5MBPbIqV92AaeXatLxBI9gBaebbnrfifHhDYfgasaacH8akY=wiFfYdH8Gipec8Eeeu0xXdbba9frFj0=OqFfea0dXdd9vqai=hGuQ8kuc9pgc9s8qqaq=dirpe0xb9q8qiLsFr0=vr0=vr0dc8meaabaqaciaacaGaaeqabaqabeGadaaakeaadaWadaqaaGGaciab=f7aHbGaay5waiaaw2faamaaDaaaleaacqaIYaGmcqaIWaamaeaacqWGebaraaaaaa@3362@ of the three saccharides was measured. The value for saccharide **1 **was +79.5, while no value was obtained for saccharides **2 **and **3**. This is due to the small quantity of saccharides **2 **and **3 **obtained, and not enough to assess this value. The degrees of polymerization were confirmed as being 3 (saccharides **1**), 4 (saccharides **2**) and 5 (saccharides **3**), as shown by measurements of [M+Na]^+ ^ions (m/z: 497, **1**: 659, **2**: 821, **3**) using TOF-MS, and analysis of the molar ratios of D-glucose, D-xylose and D-fructose.

From the GC analysis, the relative retention times of the methanolysates of the permethylated saccharides were investigated [*t*_R_, retention time of methyl 2, 3, 4, 6-tetra-*O*-methyl-β-D-glucoside = 1.00 (*t*_R_, 9.05 min)]. The methanolysate of permethylated saccharide **1 **showed four peaks corresponding to methyl 2, 3, 4, 6-tetra-*O*-methyl-D-glucoside (*t*_R_, 1.03 and 1.47), methyl 1, 3, 4, 6-tetra-*O*-methyl-D-fructoside (*t*_R_, 1.03 and 1.27) and methyl 3, 4-di-*O*-methyl-D-xyloside (*t*_R_, 1.47). Furthermore, the methanolysate of permethylated saccharide **2 **and **3 **exhibited four peaks, which corresponded to the same methyl glycosides as those observed for saccharide **1 **and two peaks corresponding to methyl 3, 4, 6 tri-*O*-methyl-D-glucoside (*t*_R_, 2.86 and 3.45). The area of peaks corresponding to the methyl glycosides obtained from the methanolysate of permethylated saccharide **3 **were larger than those of permethylated saccharide **2**. The peak area of methyl 3, 4, 6 tri-*O*-methyl-D-glucoside indicating 1→2 glucosyl linkage of each saccharide, was increased by additional units of glucose.

**Figure 2:**
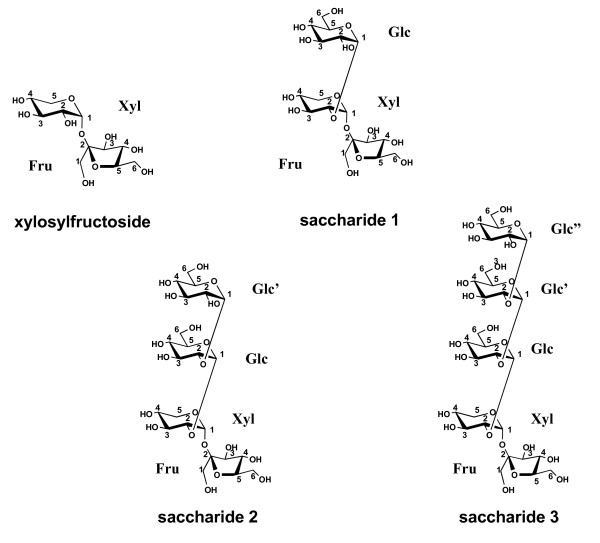
Structures of xylosylfructoside, and saccharides **1**, **2 **and **3 **synthesized by kojibiose phosphorylase.

From these findings, saccharides **1**, **2 **and **3 **were characterized as 2-α-D-glucopyranosyl-α-D-xylopyranosyl-β-D-fructofuranoside, 2(2-α-D-glucopyranosyl)_2_-α-D-xylopyranosyl-β-D-fructofuranoside, and 2(2-α-D-glucopyranosyl)_3_-α-D-xylopyranosyl-β-D-fructofuranoside, respectively (cf. Figure 2).

### Strategy for NMR analysis

The glucose, xylose and fructose residues of the synthesized saccharides are represented as, Glc: glucopyranosyl, Glc': glucopyranosyl', Glc": glucopyranosyl", Xyl: xylopyranosyl and Fru: fructofuranosyl as shown in Figure 2. The proton and carbon positions in a particular residue are represented by H-1-Glc and C-1-Xyl, respectively.

The basic strategy for the assignment of the ^1^H and ^13^C NMR signals of each compound is as follows: one anomeric position for the xylose residue; one, two or three anomeric positions of glucose residues; and one quaternary carbon for the fructose residue in each saccharide molecule. The starting point for the assignment is the anomeric protons. The two-dimensional (2D) ^1^H-^1^H COSY spectrum [9,10] reveals the connectivities of the protons in each spin system from H-1 to H-5 in the xylose and H-1 to H-6 in the glucose residues. The results from HSQC [11] and HSQC-TOCSY [12] then enable the assignment of the carbons attached to those protons and the separation of the carbon and proton of each saccharide. The HMBC spectra are also used to confirm the intra-residual assignments in the region where the protons cannot be assigned by ^1^H-^1^H COSY owing to signal overlapping [13,14]. With regard to the fructose units, one particular quaternary carbon (C-2) should be correlated with hydroxymethine protons at H-3 or H-4 by HMBC.

The proton network of Fru from H-3 to H-6 can be assigned by ^1^H-^1^H COSY and the attached carbons by HSQC. The residual H-1 and C-1 can be correlated with C-2 or C-3 and H-3, respectively, by HMBC.

The inter-residual HMBC correlation peaks between H-1-Xyl and C-2-Fru, and H-1-Glc and C-2-Xyl determined the attachment of Fru to C-1 of Xyl and the Xyl to C-1 of Glc. The linkages of one, two and three glucose residues were identified with the inter-residual H-1-Glc'/C-2-Glc and H-1-Glc"/C-2-Glc' correlation peaks in the HMBC spectrum.

Finally, the coupling patterns of overlapped ^1^H signals were analyzed using SPT (selective population transfer) experiment.

The HSQC spectrum was unhelpful in the assignment of these methylene signals, since the chemical shift difference between the C-6 carbons of interest is very small. Therefore, the resolution enhancement of the 2D HSQC method could be achieved by CH_2_-selected editing (E)-HSQC in which the ^13^C spectral width was limited in the range of the methylene carbons. This enabled sufficient ^13^C resolution to separate each CH_2 _signal of H-5/C-5 in the xylose residue, and H-6/C-6 in the glucose and fructose residues, thus leading to the unambiguous assignment of the methylene proton's chemical shift.

### Xylosylfructoside

The 1D ^1^H and ^13^C NMR spectra of xylosylfructoside showed anomeric proton (δ_H _5.34 ppm, *d*, 3.7 Hz) and carbon (δ_C _93.17 ppm) signals for the xylopyranosyl residue. The ^1^H-^1^H coupling constant value between H-1-Xyl (δ_H _5.34 ppm, d, *J *= 3.7 Hz) and H-2-Xyl (δ_H _3.53 ppm, dd, *J *= 10.0 and 3.7 Hz) determined the α-form of the glycosyl bond. The ^1^H-^1^H-COSY and the HSQC spectra completed the assignment of the protons and carbons from H-1-Xyl to H-4-Xyl. The inter-residual HMBC correlation between H-1-Xyl (δ_H _5.34 ppm) and C-2-Fru (δ_C _104.55 ppm) determined the attachment between C-2-Fru and C-1-Xyl.

**Figure 3:**
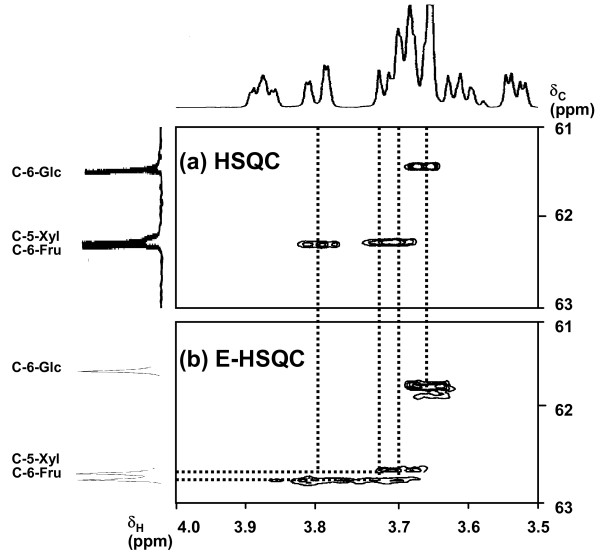
Conventional HSQC spectrum of xylosylfructoside (a). Methylene selected E-HSQC spectrum of xylosylfructoside (b).

Since the methylene proton signals of H-6-Fru and H-5-Xyl buried in the overlapped region were not separated by conventional HSQC (cf. Figure 3a), the CH_2_-selected E-HSQC spectrum of fructosylxyloside was used (cf. Figure 3b). In this spectrum, each correlation peak was well separated, and thus the chemical shift of each methylene proton was determined. Finally, the coupling patterns of the overlapped ^1^H signals were analyzed by SPT experiment.

### Saccharide 1

The 1D ^1^H and ^13^C NMR spectra of saccharide **1 **showed two anomeric proton (δ_H _5.11 ppm, *d*, 3.8 Hz and δ_H _5.59 ppm, *d*, 3.5 Hz) and carbon (δ_C _97.52 ppm and δ_C _90.84 ppm) signals for the xylopyranosyl and glucopyranosyl residues. The ^1^H-^1^H coupling constant between H-1 (δ_H _5.59 ppm, d, *J *= 3.5) and H-2 (δ_H _3.65 ppm, d, *J *= 9.8 and 3.5 Hz) of Xyl, and H-1 (δ_H _5.11 ppm, d, *J *= 3.8 Hz) and H-2 (δ_H _3.56 ppm, d, *J *= 10.1, 3.8 Hz) of Glc determined the α-form of the glycosyl bond. The ^1^H NMR spectrum showed a complex pattern in the region of δ_H _3.4–4.0 ppm. The ^1^H-^1^H-COSY and the HSQC spectra completed the assignment of the protons and carbons from H-1-Glc to H-6-Glc and from H-1-Xyl to H-4-Xyl. The inter-residual HMBC correlation between H-1-Xyl (δ_H _5.59 ppm) and C-2-Fru (δ_C _104.91 ppm), and H-1-Glc (δ_H _5.11 ppm) and C-2-Xyl (δ_C _76.39 ppm) indicated that C-2 of Fru and that of Xyl are attached to C-1-Xyl and C-1-Glc, respectively.

**Figure 4:**
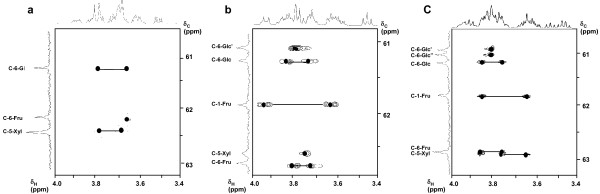
Methylene selected E-HSQC spectra of saccharides **1 **(a), **2 **(b) and **3 **(c).

Unambiguous assignments of the methylene protons H-6-Fru and H-5-Xyl buried in the overlapped region were achieved using the CH_2_-selected E-HSQC of saccharide **1 **(cf. Figure 4a). In this spectrum each correlation peak was well separated, and thus the chemical shift of each methylene proton was determined. Finally, the coupling patterns of the overlapped ^1^H signals were analyzed by SPT experiment.

### Saccharide 2

The assignment of saccharide **2 **was also started from the three anomeric protons of xylopyranosyl residue (δ_H _5.60 ppm, *d*, 3.6 Hz) and glucopyranosyl residues (δ_H _5.22 ppm, *d*, 3.5 Hz and δ_H _5.06 ppm, *d*, 3.7 Hz). The ^1^H-^1^H coupling constant between H-1 (δ_H _5.10, d, *J *= 3.7 Hz) and H-2 (δ_H _3.59, d, *J *= 3.7, 10.4 Hz) of Glc' determined the α-form of the glucosyl bond. The ^1^H-^1^H-COSY spectrum revealed the connectivities of protons from H-1 to H-4 of Glc, H-1 to H-5 of Glc' and H-1 to H-3 of Xyl, and the carbons attached to those protons were assigned from the HSQC spectrum. The inter-residual HMBC correlation between H-1-Glc' (δ_H _5.06 ppm) and C-2-Glc (δ_C _77.64 ppm) determined the connectivity between the two sugar moieties.

The assignments of the methylene protons H-5-Xyl, H-6-Fru and H-6-Glc were performed with the CH_2_-selected E-HSQC of saccharide **2 **(cf. Figure 4b). Four carbons, C-6-Glc, C-6-Glc', C-5-Xyl and C-6-Fru could be separated in the CH_2_-selected E-HSQC spectrum. Moreover, ^1^H-^1^H coupling patterns of ^1^H signals were extracted from SPT difference spectra.

### Saccharide 3

The assignment of saccharide **3 **was also begun from the three anomeric protons of the xylopyranosyl residue (δ_H _5.53 ppm, *d*, 3.2 Hz) and that of the glucopyranosyl residues (δ_H _5.32 ppm, *d*, 3.4 Hz, δ_H _5.28 ppm, *d*, 3.2 Hz and δ_H _5.10 ppm, *d*, 3.8 Hz), and carried out in the same manner as for saccharide **2**. The inter-residual HMBC correlation between H-1-Glc" (δ_H _5.10 ppm) and C-2-Glc' (δ_C _77.54 ppm) determined the connectivity between the two sugar moieties.

The assignments of the methylene protons H-5-Xyl, H-6-Fru and H-6-Glc buried in the overlapped region were achieved from the CH_2_-selected E-HSQC of saccharide **3 **(cf. Figure 4c). Five carbons (C-6-Glc, C-6-Glc', C-6-Glc", C-5-Xyl and C-6-Fru) could be separated in the CH_2_-selected E-HSQC spectrum.

The assignments of all ^1^H and ^13^C signals of saccharides **1, 2 **and **3 **are shown in Table 1 [see additional file 1].

The three saccharides formed by glucosyltransfer from β-D-G1P to fructosyl xyloside using *Thermoanaerobacter brockii *kojibiose phosphorylase were confirmed as being oligosaccharides, *O*-α-D-glucopyranosyl-(1→[2-*O*-α-D-glucopyranosyl-1]_*m*_→2)-*O*-α-D-xylopyranosyl-(1→2)-β-D-fructofuranoside: *m *= 0 (**1**), 1 (**2**) and 2 (**3**).

## Conclusion

By using kojibiose phosphorylase, three novel oligosaccharides have been synthesized. These saccharides were purified and their structure was fully determined.

## Experimental

### Saccharides

Crystalline xylosylfructoside [*O*-α-D-xylopyranosyl-(1→2)-β-D-fructofuranoside] was prepared from sucrose and D-xylose by *Scopulariopsis brevicaulis *[8]. Kojibiose and β-D-glucose 1-phosphate (β-D-G1P) were purchased from Sigma Chemical Co. (St. Louis, MO, USA).

### Enzyme

Kojibiose phosphorylase was purified from a cell-free extract of *T. brockii ATCC 35047 *[1].

### High performance anion-exchange chromatography (HPAEC)

The oligosaccharides were analyzed using a Dionex Bio LC Series apparatus equipped with an HPLC carbohydrate column (Carbo Pack PA-1, inert styrene divinylbenzene polymer) and a pulsed amperometric detector (PAD) [15,16]. The mobile phase consisted of eluent A (150 mM NaOH) and eluent B (500 mM sodium acetate in 150 mM NaOH) with a sodium acetate gradient as follows: 0–1 min, 25 mM; 1–2 min, 25–50 mM; 2–20 min, 50–200 mM; 20–22 min, 500 mM; 22–30 min, 25 mM; using a flow rate of 1.0 mL/min. The applied PAD potentials for E1 (500 ms), E2 (100 ms), and E3 (50 ms) were 0.1, 0.6, and -0.6 V [17] respectively, and the output range was 1 μC.

### Isolation of the oligosaccharide synthesized from xylosylfructoside and β-D-G1P by kojibiose phosphorylase

The reaction mixture (25.0 mL) containing kojibiose phosphorylase (4.0 units), xylosylfructoside (500 mg), β-D-G1P (500 mg) and acetate buffer (0.05 M, pH 5.5), was incubated at 50°C for 54 h. The reaction was terminated by heating the mixture in a boiling water bath for 5 min. The reaction mixture was then loaded onto a carbon-Celite [1:1; charcoal (Wako Pure Chemical Industries, Ltd., Osaka, Japan) and Celite-535 (Nakarai Chemical Industries, Ltd., Osaka, Japan)] column (6.0 × 45 cm) and successively eluted with water, 5% EtOH (79 L), 10% EtOH (17 L), 13% EtOH (8 L) and 15% EtOH (16 L). The 10%, 13% and 15% EtOH fractions contained a mixture of saccharides **1, 2 **and **3**, respectively. The three fractions were then purified using preparative HPLC. Portions of 10%, 13% or 15% EtOH fractions were applied to an HPLC system (JASCO GULLIVER, Tokyo, Japan) equipped with an ODS column (TSKgel ODS-80Ts, 20 mm × 25 cm, Tosoh, Tokyo, Japan) at 35°C, and eluted with water at a flow of 3.5 mL/min, using a refractive index detector. Saccharides **1 **(96.0 mg), **2 **(47.5 mg) and **3 **(29.4 mg) were obtained by the repeated HPLC purification procedure.

### Matrix assisted laser desorption ionization time of flight mass spectrometry (MALDI-TOF-MS)

MALDI-TOF-MS spectra were obtained on a Shimadzu-Kratos mass spectrometer (KOMPACT Probe) using 2, 4-dihydroxybenzoic acid matrix.

### Methylation and methanolysis

Methylation of the oligosaccharides was carried out according to the Hakomori method [18]. The permethylated saccharides were methanolysed by heating in 1.5% methanolic hydrochloric acid at 96°C for 10 or 180 min. The reaction mixture was treated with Amberlite IRA-410 (OH^-^) to remove hydrochloric acid, and dried under vacuum. The resulting methanolysate was dissolved in a small volume of methanol and analyzed using gas chromatography.

### NMR measurements

Xylosylfructoside and each oligosaccharide **1**, **2 **or **3 **(10 mg) were dissolved separately in 0.5 mL D_2_O. NMR spectra were recorded at room temperature with a Bruker AMX 500 spectrometer (^1^H 500 MHz, ^13^C 125 MHz) equipped with a 5 mm diameter C/H dual (1D spectra) and TXI probe (2D spectra). Chemical shifts of ^1^H (δ_H_) and ^13^C (δ_C_) in ppm were determined relative to the external standard of sodium [2, 2, 3, 3-^2^H_4_]-3-(trimethylsilyl) propanoate in D_2_O (δ_H _0.00 ppm) and 1, 4-dioxane (δ_C _67.40 ppm) in D_2_O, respectively.

### 1D nomal ^1^H and ^13^C spectra

1D ^1^H and ^13^C spectra were recorded with 32 K data points for a spectral width of 8064 Hz at 500.133 MHz (^1^H) and with 64 K data points for a spectral width of 33333 Hz at 125.772 MHz (^13^C). Exponential multiplication (LB = 0.2 for ^1^H and 1.0 for ^13^C) was performed prior to Fourier transformation. For the ^13^C spectrum, complete proton decoupling was derived by attenuation of the high-power output of the decoupler (p/2 pulse duration 100 ms). For the SPT spectrum, selective irradiation was performed by attenuation of the low-power of the decoupler (115 dB) for 2 s.

### ^1^H-^1^H COSY spectra

The ^1^H-^1^H COSY spectra were measured with a relaxation delay of 1.9 s covering a spectral width of 2762 Hz in both dimensions with 1024 K data points using one, one and four transients for each of the 256 *t*_1 _increments [9,10]. Zero-filling to 512 for *F*_1 _and multiplication with a sine-bell window in both dimensions were performed prior to 2D Fourier transformation. The total measuring times for xylosylfructoside, saccharides **1**, **2 **and **3 **were ca. 9, 9, 9 and 36 min, respectively.

### HSQC spectra

The gradients selected for HSQC spectra covering a spectral width of 2762 and 6666 Hz in both dimensions were measured with 1024 data points using four transients for each of the 512 *t*_1 _increments [11]. The relaxation and evolution delays [1/4 ^1^*J *(C, H)] were set to 2.0 s and 1.9 ms, respectively. Zero-filling to 1024 for *F*_1 _and multiplication with a squared sine-bell shifted by π/2 for *F*_2 _and π/6 for *F*_1 _windows in both dimensions were performed prior to 2D Fourier transformation. The total measuring times for xylosylfructoside, saccharides **1**, **2 **and **3 **were ca. 50, 78, 78 and 78 min each.

### HSQC-TOCSY spectra

The phase-sensitive HSQC-TOCSY spectra were determined by the sequence including inversion of direct resonance (IDR). The TOCSY mixing for 264 ms was composed of MLEV-17 composite pulses guarded by trim pulses (2.5 ms) derived from the high-power output of the ^1^H pulse attenuation by 14 dB (π/2 pulse duration, 40 μs). The delays for relaxation and evolution [1/4 ^1^*J *(C, H)] were set to 2.1 s and 1.8 ms respectively. The HSQC-TOCSY spectra of **1**, **2 **and **3 **were measured using the sequence covering a spectral width of 2762 Hz in *F*_2 _and 6667, 6849 and 6667 Hz in *F*_1 _with 1024 data points using 32, 32 and 64 transients for each of the 512, 460 and 512 *t*_1 _increments. Zero-filling to 1024 for *F*_1 _and multiplication with a sine-bell windows shifted by π/2 for *F*_2 _and π/2, π/6 and π/2 for *F*_1 _and in both dimensions were performed prior to 2D Fourier transformation. The total measuring times for saccharides **1**, **2 **and **3 **were circa 12, 11 and 23 h, respectively.

### HMBC spectra

The HMBC spectra were obtained using the pulse sequence of CT-HMBC 2 proposed by Furihata and Seto [14]. The HMBC spectra of xylosylfructoside, saccharides **1**, **2 **and **3 **were measured by the sequence covering a spectral width of 2762 Hz in *F*_2 _and 6667 Hz in *F*_1 _with 1024 data points using 4, 32, 48 and 64 transients for each of the 512 *t*_1 _increments. Zero-filling to 1024 for *F*_1 _and multiplication with a Lorenz-Gaussian window (GB = 0.5, LB = -2) in *F*_2 _and multiplication with a sine-bell shifted by π/8 window *F*_1 _were performed prior to 2D Fourier transformation. The delays for relaxation, low-pass *J*-filter [1/2 ^1^*J *(C, H)] and evolution [1/2 ^LR^*J *(C, H)] were set to 1.7 s, 3.5 ms and 80 ms, respectively. The total measuring times for xylosylfructoside, saccharides **1**, **2 **and **3 **were ca. 1, 9, 13 and 18 h, respectively.

### CH_2_-selected E-HSQC

The CH_2_-selected E-HSQC spectra of xylosylfructoside, saccharides **1**, **2 **and **3 **were measured by the sequence covering a spectral width of 2762 Hz in *F*_2 _and 352, 353, 323 and 353 Hz in *F*_1_. For the CH_2_-selected E-HSQC spectra, 4, 8, 8 and 32 transients were accumulated with a total measuring time of ca. 0.7, 1, 1 and 5 h, respectively. The other measuring conditions were the same as these used for the of HSQC spectra.
